# The identification of unique serum proteins of HIV-1 latently infected long-term non-progressor patients

**DOI:** 10.1186/1742-6405-7-21

**Published:** 2010-07-06

**Authors:** Rachel Van Duyne, Irene Guendel, Kylene Kehn-Hall, Rebecca Easley, Zachary Klase, Chenglong Liu, Mary Young, Fatah Kashanchi

**Affiliations:** 1 The George Washington University Medical Center, Department of Microbiology, Immunology, and Tropical Medicine, Washington, DC 20037, USA; 2 George Mason University, Department of Molecular and Microbiology, National Center for Biodefense & Infectious Diseases, Manassas, VA 20110, USA; 3 Molecular Virology Section, Laboratory of Molecular Microbiology, NIAID, National Institutes of Health, Bethesda, Maryland 20892-0460, USA; 4 Washington Metropolitan Women's Interagency HIV Study, Division of Infectious Diseases, Georgetown University Medical Center, Washington, DC 20007, USA; 5 National Center for Biodefense and Infectious Diseases Professor of Microbiology George Mason University Discovery Hall, Room 306 10900 University Blvd. MS 1H8 Manassas, VA 20110, USA

## Abstract

**Background:**

The search for disease biomarkers within human peripheral fluids has become a favorable approach to preventative therapeutics throughout the past few years. The comparison of normal versus disease states can identify an overexpression or a suppression of critical proteins where illness has directly altered a patient's cellular homeostasis. In particular, the analysis of HIV-1 infected serum is an attractive medium with which to identify altered protein expression due to the ease and non-invasive methods of collecting samples as well as the corresponding insight into the *in vivo *interaction of the virus with infected cells/tissue. The utilization of proteomic techniques to globally identify differentially expressed serum proteins in response to HIV-1 infection is a significant undertaking that is complicated due to the innate protein profile of human serum.

**Results:**

Here, the depletion of 12 of the most abundant serum proteins, followed by two-dimensional gel electrophoresis coupled with identification of these proteins using matrix-assisted laser desorption/ionization time-of-flight (MALDI-TOF) mass spectrometry, has allowed for the identification of differentially expressed, low abundant serum proteins. We have analyzed and compared serum samples from HIV-1 infected subjects who are being treated using highly active antiretroviral therapy (HAART) to those who are latently infected but have not progressed to AIDS despite the absence of treatment, i.e. long term non-progressors (LTNPs). Here we have identified unique serum proteins that are differentially expressed in LTNP HIV-1 patients and may contribute to the ability of these patients to combat HIV-1 infection in the absence of HAART. We focused on the cdk4/6 cell cycle inhibitor p16^INK4A ^and found that the treatment of HIV-1 latently infected cell lines with p16^INK4A ^decreases viral production despite it not being expressed endogenously in these cells.

**Conclusions:**

Identification of these unique proteins may serve as an indication of altered viral states in response to infection as well as a natural phenotypic variability in response to HIV-1 infection in a given population.

## Background

Human serum is derived from the liquid plasma component of the blood with the fibrinogens, or clotting factors, removed and is composed of small molecules such as salts, lipids, amino acids, sugars and approximately 60-80 mg of proteins/mL [1]. Serum is a readily obtainable peripheral bodily fluid from which the protein profile directly reflects the normal or disease state of the organism [2-4]. Serum is a complex mixture of "classical" and "non-classical" proteins. Classical serum proteins are involved in a number of processes including proteolysis, inhibition, binding, transport, coagulation, and immune response and are often secreted from the liver, through the intestines, and into the bloodstream [5]. "Non-classical" proteins are proteins that are not directly tied to any known function within the serum and often originate from cellular leakage or shedding, and may utilize the bloodstream for transportation [5]. It is generally accepted that most of the significant changes in the serum will be found in these low abundant non-classical proteins, due to the hypothesis that the presence of these proteins should reflect changes in the diseased tissue. Indeed, serum commonly contains upwards of 10,000 different proteins at any given time that are being actively produced and secreted by all cells and tissues, therefore, the proteomic profile of serum can give insight into the systemic reaction to a disease state and can serve as a pool of differentially expressed proteins [2,6-13]. Recently, the interest in characterizing the human serum proteome has increased due to the determination of disease biomarkers for early detection, diagnosis, and drug targeting; however, due to the extensive dynamic range of protein concentration within the serum, the identification of low abundance proteins suitable for biomarker determination is often masked. The 22 highly abundant proteins contained within serum constitute approximately 99% of the total serum proteins, including albumin, IgG, transferrin, haptoglobin, fibrinogen, etc. and interfere with the identification of low abundance proteins in the ng/mL concentration range. The presence of these highly abundant proteins necessitates the prefractionation of serum samples prior to analysis for low abundant proteins. Due to the dynamic insight the analysis of the serum proteome can relate to a disease state, of particular interest is the identification of low abundant proteins that change in expression or abundance in response to a disease state. These low abundant proteins could potentially arise as an early diagnostic for a disease state, or a therapeutic target.

Serum proteomics has emerged as an integral biomarker identification and diagnostic tool, especially for infectious diseases and oncology. Recently, novel serum biomarkers have been identified for liver fibrosis in hepatitis C virus (HCV) infected patients as well as unique protein signatures in SARS coronavirus infections, and infant hepatitis syndrome induced by human cytomegalovirus (HCMV) infection [14-16]. Characterization of the serum protein profile of these viral states helps provide insight into the expression changes associated with viral infection. In particular, HIV-1 infection, even at the acute phase, results in dramatic changes in both cellular and viral protein expression levels. As the HIV-1 viral tropism consists primarily of CD4+ T-cells, macrophages, and dendritic cells, the resulting protein changes can be seen systemically as infected cells travel throughout the body. Additionally, the nature of this viral infection supports the secretion of altered proteins into the blood and subsequently the serum due to the propensity of the virus to stimulate apoptosis of infected cells, therefore emptying cellular contents into the serum. These characteristics of HIV-1 infection suggest that the analysis of the serum of infected patients is an appropriate reflection of a patients' altered protein expression state.

Due to innate genetic and phenotypic differences in the human population, significant variability exists in the susceptibility to HIV-1 infection. Amongst this diversity includes the well-studied CCR5Δ32 inherited mutation, which prevents the binding of R5-tropic HIV-1 strains to the CCR5 chemokine receptor on the surface of CD4+ T-cells, therefore preventing entry of the virus [17]. Additionally, some individuals can be infected with HIV-1, however will not progress to AIDS even in the absence of therapy. These Long Term Non-Progressors (LTNPs) are often characterized as being infected with HIV-1 but are also disease free and sustain a normal CD4 T-cell count and a low viral load. Over the past 20 years, multiple studies have been aimed at determining the reason that these individuals are able to resist disease progression. There are studies that suggest that the virus infecting these cells could be deficient in some way, for example, Nef deficient viruses and Vpr R77Q mutations are associated with LTNPs [18-22]. A number of host factors have also been identified that may contribute to the observed resistance. LTNPs have a higher prevalence of the CCR5Δ32 allele [17,23-25]. In addition, the presence of certain HLA genes including HLA-B27, HLA-B*5701, HLA-B*5401, and HLA-B*1507 have been linked to LTNP [26-28] however, the identified alterations do not account for all cases of LTNP. Therefore, the search for protective host factors is still an area of active investigation in hopes of obtaining information that could be of therapeutic value.

Here, we describe the detection of unique, low abundant serum proteins in latently infected HIV-1 LTNPs as compared to serum from patients undergoing HAART treatment, and those not infected with HIV-1. We attempted to characterize the underlying differences in LTNPs that contribute to the ability of these patients to combat HIV-1 infections. We have depleted 12 of the most highly abundant serum proteins from three sets of serum samples (uninfected, infected on HAART, LTNP) and identified differentially expressed proteins across the samples. In particular, we focus on the identified cellular protein p16^INK4A ^which is found preferentially in LTNP patient serum samples, but is not present in patients undergoing HAART treatment. *In vitro *viral assays and viability studies confirm the loss of viral replication upon p16^INK4A ^treatment in latently infected cell lines and the non-toxic effect of the same treatment in corresponding uninfected cell lines.

## Results

### Depletion of the 12 highly abundant serum proteins allows for the identification of low abundant proteins

**Figure 1:**
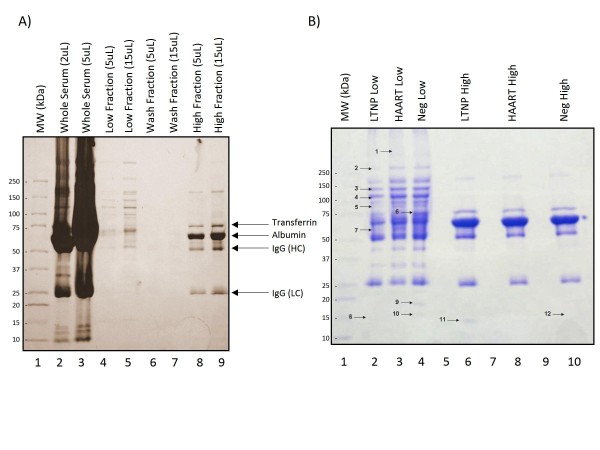
**1D Demonstration of the depletion capabilities of the IgY-12 High Capacity SC Spin Column kit on patient serum**. Depletion of patient serum was performed as indicated by manufacturer's instructions. Low and High abundant fractions were collected for each sample and run on a 1D 4-20% Tris-Gycine SDS-PAGE gel. **A) **Whole serum (lanes 2, 3) was incubated with the column containing antibodies against 12 of the high abundant serum proteins. Low abundant proteins (lanes 4, 5) were collected as the flowthrough, the column was washed (lanes 6, 7) and the high abundant proteins eluted (lanes 8, 9). Briefly the observed high abundant proteins were compared to the known sizes of the expected proteins as indicated. **B) **Equal volumes of serum from each of the six patients within each category (LTNP, HAART, and Negative) were pooled together to create a stock of each condition, independent of patient-to-patient variability. Twenty microliters of each stock was subjected to depletion and equal concentration of Low and High fraction were run on a 1D gel. Lanes 2, 3 and 4 are the low abundance fractions of the pooled LTNP, HAART, and Negative patients, respectively. Lanes 6, 8, and 10 are the high abundance fractions of the pooled LTNP, HAART, and Negative patients, respectively. The indicated arrows represent differentially expressed proteins that were excised, trypsinized, and identified using MALDI-TOF for preliminary protein screening.

To begin the identification of unique serum proteins, we obtained 18 subject serum samples: six LTNP, six HIV-1 infected subjects receiving HAART therapy (HAART) and six HIV-uninfected individuals through the Washington DC site of the Women's Interagency HIV Study (WIHS) Georgetown site (Table 1). WIHS is an NIH multicenter study of the natural history of HIV-1 infection in women [29]. LTNPs are defined by WIHS as being HIV-1 infected, but disease free for at least five years, having a CD4 count of greater than 500 at all visits and having no history of anti-retroviral therapy. The difficulty associated with analyzing serum is the presence of a high abundance of proteins which mask potential low abundance biomarkers. To overcome this obstacle, we utilized the ProteomeLab IgY serum depletion kit which removes 12 of the most abundant proteins in serum: albumin, IgG, transferrin, fibrinogen, IgA, α2-macroglobulin, IgM, α1-antitrypsin, haptoglobin, α1-acid glycoprotein, apolipoprotein A-I, and apolipoprotein A-II. As can be observed in Figure 1A, whole serum (lanes 2, 3) contains many proteins and is too complex to allow for confident identification of specific proteins. However, when the high abundant proteins (Figure 1, lanes 8, 9) are removed, lower abundant proteins that were originally masked (Figure 1, lanes 4, 5) are able to be analyzed. Along these lines, we found the ProteomeLab IgY serum depletion kit to be the most appropriate and reproducible manner in which to fractionate our serum samples into high and low abundant fractions. We applied this depletion strategy to pooled patient samples, combining equal volumes of whole serum from each of the six patients per sample set (LTNP, HAART, and Negative), which were subsequently depleted into low and high abundance fractions. We began the analysis with pooled samples to assist in the identification of HIV-1 infection specific protein identification as opposed to identifying individual patient and serum variability. These pooled samples were separated based on 1D SDS-PAGE (Figure 1B) and comparisons between LTNP, HAART, and Negative low abundant samples were carried out via in-gel trypsin digestion, peptide elution and desalting, followed by MALDI-TOF mass spectrometry as indicated by numbered arrows marking excised bands. The subsequent protein identifications served as a preliminary indication of differentially expressed proteins between the three patient types. These observations, as summarized in Table 2, provide an insight into the relevance of proteins identified in the context of the state of HIV-1 infection. Of particular interest in Table 2 is the identification of HIV-1 enhancer binding protein 1, (HIVEP1), Ribonuclease III, and heterochromatin protein 1 binding protein in the low abundance LTNP fraction. HIVEP1 is a member of the ZAS family of proteins which bind the promoter and enhancer regions of both cellular genes and infectious viruses, including HIV-1. Also known as PRDII-BF1 or MBP-1, this transcription factor binds to both the NF-κB and the TAR transactivation response DNA elements on the HIV-1 LTR in both the presence and absence of HIV-1 Tat [30,31]. It is not surprising that a transcription factor such as HIVEP1 would be present during HIV-1 infection; however, the identification of this protein is not necessarily a marker for a LTNP phenotype. Ribonuclease III, or Drosha, is a cellular enzyme found in the nucleus which serves to cleave double-stranded RNA hairpin transcripts as a key step in the production of miRNAs in the RNA interference pathway. Interestingly, heterochromatin protein 1, or HP1 is a member of the chromatin remodeling family of proteins, which can bind histones at methylated lysine residues and can interact with many chromatin-associated nonhistone proteins. The HP1 family of proteins has been associated with promoting a heterochromatic cellular state, where latently HIV-1 infected cells can persist as a transcriptionally silent provirus [32,33]. It may be of interest that an HP1 binding protein would be present in the serum of an HIV-1 infected patient as HP1, including its subtypes α, β, and γ, could be involved in the control of various stages of infection. It is possible that the association of this HP1 binding protein with varying subtype of HP1 could explain the differences in patient phenotypes, especially those that result in an altered susceptibility to viral infection.

**Table 1:** Patient samples obtained from the WIHS Interagency Cohort.

Ref. #	Group	Concentration (μg/μl)
**1**	LTNP	11.26

**2**	LTNP	10.80

**3**	LTNP	11.85

**4**	LTNP	11.44

**5**	LTNP	12.05

**6**	LTNP	12.12

**7**	HAART Responder	12.18

**8**	HAART Responder	12.80

**9**	HAART Responder	12.23

**10**	HAART Responder	12.12

**11**	HAART Responder	11.04

**12**	HAART Responder	11.79

**13**	Negative	10.43

**14**	Negative	11.97

**15**	Negative	12.55

**16**	Negative	12.83

**17**	Negative	12.32

**18**	Negative	11.60

**Table 2:** Protein Identification of Serum Depleted Samples from 1D SDS-PAGE

Spot #	Group	Type	Protein Name	Accession #	pI	MW (kDa)
**2**	LTNP	Low	Human immunodeficiency virus type 1 enhancer binding protein 1	gi 55662194	8.3	296.68

**3**	LTNP	Low	Ribonuclease III (Drosha)	gi 20139357	8.5	159.23

**4**	LTNP	Low	ADAM metallopeptidase with thrombospondin type 1 motif, 18	gi 38649249	9.7	135.12

**5**	LTNP	Low	Tax1-binding protein TXBP151	gi 5776545	5.3	86.23

**7**	LTNP	Low	Heterochromatin protein 1, binding protein	gi 55961949	9.8	57.19

**8**	LTNP	Low	Gga-Vhs domain & Beta-Secretase C-terminal phosphopeptide	gi 38492866	5.5	17.92

**11**	LTNP	High	MHC class I antigen	gi 33413287	8.0	10.43

**1**	HAART	Low	Coagulation factor V (Proaccelerin, labile factor)	gi 56417672	5.7	252.19

**6**	Uninfected	Low	Matrix metalloproteinase 2 preprotein	gi 11342666	5.3	73.86

**9**	Uninfected	Low	Ribosomal protein L27	gi 4506623	10.6	15.78

**10**	Uninfected	Low	Ribosomal protein L36a	gi 10445223	11.1	12.42

**12**	Uninfected	High	P63 protein	gi 34304700	7.1	11.39

### 2DGE and MALDI-TOF analysis of pooled, depleted serum samples identified unique low abundance proteins

**Figure 2:**
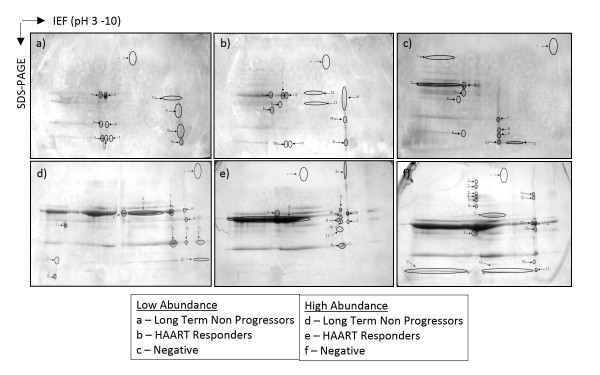
**Two-dimensional gel electrophoresis of pooled patient samples**. Post sample depletion, six 2D gels (IPG Strip pH 3-10, 4-20% SDS-PAGE) were run in tandem to separate the low and high abundance protein fractions of pooled patient serum samples in tandem. Gels a, b, and c are representative of LTNP, HAART, and Negative low abundance patient samples respectively. Gels d, e, and f are representative of LTNP, HAART, and negative high abundance patient samples respectively. Arrows and circles indicate protein spots excised for MALDI-TOF analysis.

Following the initial 1D separation and MALDI-TOF MS assisted identification, the pooled patient samples were subjected to 2D-gel electrophoresis (2DGE); isoelectric focusing (IEF) using IPG strips with a pH 3.0-10.0 range followed by SDS-PAGE using 4-20% Tris-Glycine Criterion gels. The method of 2D-gel electrophoresis is much more sensitive than 1D-gel electrophoresis in that it provides separation of a complex mixture of proteins in two dimensions, therefore removing the complexity associated with overlapping proteins, or masking due to post-translational modifications. 2DGE is a more sensitive front-end purification approach to the isolation and identification of individual protein species by mass spectrometry. Figure 2 depicts the LTNP, HAART, and Negative low abundance fractions in gels "a", "b", and "c", respectively, as well as the LTNP, HAART, and Negative high abundance fractions in gels "d", "e", and "f", respectively. Indicated protein spots from all gels were excised based on a comparison of protein abundance and the presence of unique spots in a given patient set, were subjected to in-gel trypsin digestion, and were identified by MALDI-TOF mass spectrometry. It is important to note that although gels "d," "e," and "f" contain the majority of the high abundance proteins, unique small protein spots can still be visualized on these gels. This indicates that not only will these high abundant proteins mask proteins of interest; they can also interact with and seclude lower abundance proteins from being identified. Peak lists from the collected mass spectra were processed via peptide mass fingerprinting (PMF) analysis using the Mascot and ProFound databases, compared, and compiled into a non-exhaustive list of identified proteins as displayed in Table 3. Of particular interest are those proteins identified from gel "a" indicating unique low abundance proteins in the serum of LTNP patients: Tropomyosin 3, protein kinase 3, and cdk4/6 binding protein p16. Tropomyosin interacts with actin filaments to provide stability and regulates other actin binding proteins. This family of proteins has been shown to be cleaved by HIV-1 protease *in vitro*, resulting in the dissociation of critical cytoskeletal elements, which may demonstrate the alteration of muscle structure in the presence of an HIV-1 infection [34]. Protein kinase 3, or Protein kinase C (PKC) is a member of the family of serine/threonine kinases that are integrally involved in key cellular signaling pathways and can phosphorylate a wide variety of substrates. Not surprisingly, HIV-1 infection alters the PKC phosphorylation pathway to stimulate TNF-α production by monocytes as well as other cytokines and growth factors such as IL-6, IL-10, and MCP-1 [35-39]. PKC has also been shown to be necessary for HIV-1 Tat-mediated transactivation as well as directly phosphorylating Tat at serine 46 [40,41] and plays an integral role in the signaling and secretion of cytokines in response to HIV-1 envelope proteins gp120, gp160, and gp41 [42,43]. Of particular interest in the low abundance, LTNP fraction is the presence of the cdk4/cdk6 binding protein p16, or more specifically, p16^INK4A^, a member of the inhibitor of kinase 4/alternative reading frame (INK4/ARF) family of endogenous cdk (cyclin-dependent kinase) inhibitors [44]. Dysregulation of the cell cycle, including the manipulation of cdks and their associated Cyclins is often a hallmark of cancerous and infectious phenotypes. Indeed HIV-1 and its associated proteins have been known to alter the phosphorylation state and activity of these kinases. P16^INK4A ^inhibits the phosphorylation of Rb by competitively inhibiting the association of cdk4/Cyclin D therefore inhibiting the release of Rb-bound proteins, such as E2F, and the subsequent progression into the S phase of the cell cycle [44,45]. This small molecular weight protein is an attractive candidate for a secreted, differentially expressed protein in response to HIV-1 infection.

**Table 3:** Protein Identification of Serum Depleted Samples from 2D SDS-PAGE

Spot #	Group	Type	Protein Name	Accession #	pI	MW (kDa)	% Coverage
**A2**	LTNP	Low	Serum albumin	gi 23307793	6.1	69.38	14%

**A4**	LTNP	Low	Tropomyosin 3	gi 55665778	4.8	26.26	13%

**A6**	LTNP	Low	Protein kinase 3	gi 4226043	6.4	13.48	12%

**A7**	LTNP	Low	Cyclin D-dependent kinase 4 and 6-binding protein/p16	gi 861472	5.7	16.51	24%

**B1**	HAART	Low	Serine/Threonine kinase 33	gi 23943882	6.6	57.81	14%

**B3**	HAART	Low	Kelch repeat domain containing protein 11	gi 7662260	5.8	65.7	12%

**B15**	HAART	Low	SNW1 protein/APAF1 interacting protein	gi 40850966	9.9	35.97	21%

**C2**	Uninfected	Low	Eukaryotic translation initiation factor 4B	gi 49256408	5.5	69.15	10%

**C4**	Uninfected	Low	RRBP1 protein	gi 38014595	4.9	73.67	13%

**D1**	LTNP	High	FGFR1 oncogene partner	gi 15080276	4.5	40.9	12%

**D5**	LTNP	High	Serum albumin	gi 23307793	6.1	69.38	15%

**D6**	LTNP	High	Anti-HIV-1 gp120 IgG 16c kappa light chain	gi 40647136	7.8	20.67	26%

**D7**	LTNP	High	Serum albumin	gi 23307793	6.1	69.38	19%

**D8**	LTNP	High	PCTAIRE protein kinase 3	gi 55960102	9.1	54.16	18%

**E1**	HAART	High	Pre-B-cell leukemia homeobox interacting protein 1	gi 55960102	5.2	72.9	9%

**E2**	HAART	High	Serum albumin	gi 23307793	6.1	69.38	15%

In addition to these low abundant protein identifications, the LTNP high abundant samples (gel "d") indicated the presence of the FGFR1 oncogenic partner and PCTAIRE protein kinase 3 as well as an anti-HIV-1 gp120 IgG 16 cκ light chain. FGFR1 oncogene partnered with the fibroblast growth factor receptor 1 (FGFR1) is thought to be associated with myeloproliferative disorders and as of yet is not associated with any HIV-1 protein interactions or associated disease phenotypes. PCTAIRE protein kinase 3, however, is a member of the serine/threonine family of protein kinases and more specifically, the cdc2/cdkx subfamily that plays a role in broad signal transduction pathways. This serine/threonine kinase family member has also been associated with the essential regulation of cell cycle progression, as well as transcription and DNA repair [46]. This protein identification again demonstrates the role that HIV-1 infection plays in the dysregulation of cellular kinases and specifically, cell cycle progression.

The HAART responder patient samples (gels "b" and "e") also contained unique protein candidates: serine/threonine kinase 33, the Kelch repeat domain containing protein 11, and the SNW1 protein/APAF1 interacting protein in the low abundant fraction as well as the pre-B-cell leukemia homeobox interacting protein 1 in the high abundant fraction. Of functional interest is the general serine/threonine kinase 33 as we have already identified several cellular kinases of the same family. Additionally, the SNW1 protein is a transcriptional coactivator that induces the expression of vitamin D, retinoic acid, estrogen, and glucocorticoid associated genes. SNW1/SKIP interacts with HIV-1 Tat through the association with p-TEFb (cdk9/Cyclin T1) at the TAR RNA complex, stimulating HIV-1 transcription elongation [44]. Interestingly, some of the protein spots identified the presence of serum albumin contamination (spots a2, d5, d7, and e2), which both served as an internal positive control for mass spectrometry and also indicated that the depletion columns are not completely efficient at removing contaminating high abundant proteins.

### Validation of MS protein identifications by Western Blot

**Figure 3:**
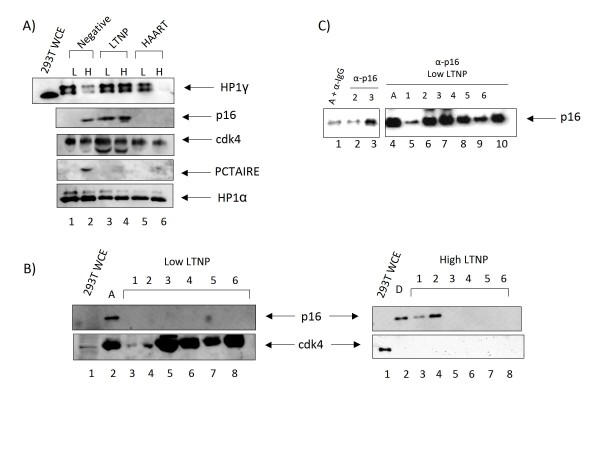
**Western blot confirmation of MALDI-TOF identified serum proteins**. **A) **Western blots were performed against pooled low (L) and high (H) abundance protein fractions for negative (lanes 2, 3), LTNP (lanes 4, 5), and HAART (lanes 6, 7) patients. Antibodies specific to cdk4, p16^INK4A^, PCTAIRE, HP1α, and HP1γ were used. **B) **Western blots were performed against individual patient samples 1-6, low and high abundant LTNPs. Antibodies specific to p16^INK4A ^and cdk4 were used. 293T, the pooled low abundance LTNP samples "A," and the pooled high abundance LTNP samples "D" were used as controls. **C) **Immunoprecipitation of p16^INK4A ^from the individual low abundant LTNP patient samples, followed by a western blot against p16^INK4A^. HeLa whole cell extract and the pooled low abundance LTNP sample "A" were used as controls.

In order to further confirm the presence of these proteins in the serum as identified by mass spectrometry, we performed western blots on the same low and high abundant pooled fractions (Figure 3A). P16^INK4A ^is present in both the low and high abundant fractions of the pooled LTNPs (lanes 3, 4) and is also observed in the high abundance fraction of uninfected patients (Figure 3A, lane 2). Interestingly, this protein is not present in HAART patient samples at all (Figure 3A, lane 5, 6). The presence of this protein in serum may be specific to individuals that confer resistance to chronic HIV-1 infection. As p16^INK4A ^is an inhibitor of cell cycle kinases, in particular cdk4 and cdk6, the levels of cdk4 in the serum samples was assayed and was shown to be ubiquitously expressed across all low and high abundance serum samples (Figure 3A, third panel from top). Indeed, levels of cdk6 were not detectable in any of the patient serum samples as compared to a 293T whole cell extract positive control (data not shown). This implies that the presence of cdk4 in the serum is not dependent on the presence or absence of p16^INK4A ^and likewise, p16^INK4A ^does not affect the expression levels of cdk4 amongst the patient serum samples. The HP1 binding protein was initially identified in the 1D/mass spectrometry analysis in the LTNP low abundance fraction, therefore the serum levels of both HP1α and HP1γ subunits were assayed (Figure 3A). The family of heterochromatin-associated proteins exist as three distinct isoforms, α, β, and γ and all act as regulators of heterochromatin-mediated transcriptional silencing [47]. HP1α has been shown to directly interact with DNA methyltransferases and histone methyltransferases to mediate transcriptional silencing [48,49] and HP1γ, in particular, interacts with the histone methyltransferase Suv39H1 to initiate a chromatin-mediated repressive state of the HIV-1 integrated virus [50]. HP1α was shown to be present in the low abundance fractions of all of the patient phenotypes whereas HP1γ was shown to be present in both the low and high abundant fractions across all patient types (Figure 3A). HP1γ is observed in lower amounts in both the Negative and HAART high abundance fractions and all serum samples indicate the presence of a post-translational modification (i.e. a doublet band) as compared to the 293T whole cell extract positive control. This indicates that the HP1γ found in serum exists in both a modified and unmodified form. Interestingly, PCTAIRE was present in the highest abundance in the uninfected (Negative), high abundance fraction (Figure 3A, lane 2), however low levels were also seen in both LTNP and HAART high abundance fractions (Figure 3A, lane 4, 6). PCTAIRE was identified initially by mass spectrometry in the high abundance LTNP sample and can be seen in the high abundance fractions of all three of the patient types biochemically, however it is present in lower amounts in the HIV-1 infected patients, indicating that this kinase may be differentially expressed upon infection though not necessarily a unique identifier for infection. P16^INK4A ^is the only protein identified from mass spectrometric analysis and confirmed biochemically that is specific for the low abundance LTNP serum samples; although the protein is also identified in the uninfected and the high abundance LTNP fractions. These results are also interesting due to the involvement of p16^INK4A ^in alterations of cell cycle control, additionally, mutations in p16^INK4A ^are found in various cancers including pancreatic, lymphomas, and sarcomas, contributing to cancer progression [45]. These findings also indicate a difference in composition of serum proteins present in HIV-1 infected individuals undergoing HAART treatment versus those that are naturally non-progressing.

In order to address the concern that the protein signature of the pooled set of samples for each patient type may not be an accurate representation of the individual variability that could be present, we screened the low abundance fractions of the LTNPs for the presence of both p16^INK4A ^and cdk4. Results in Figure 3B indicate the lack of detection of p16^INK4A ^in any of the low abundant LTNP samples, especially as compared to the pooled sample "A" (lane 2). In contrast, p16^INK4A ^was detectable in LTNP patients 1 and 2, as well as in the pooled sample "D" for the corresponding high abundance fractions (lanes 2, 3, 4). Due to the nature of the depletion step based on immuno-affinity, it is not surprising that p16^INK4A ^is detectable in individual high abundant samples, as it is probably coupled to a larger, more abundant protein and was not efficiently depleted. Additionally, post-depletion, the low abundant fractions for each patient were very dilute and the protein levels were undetectable by traditional methods. Cdk4 was detectable in the LTNP low abundant individual samples with variable abundance, correlating with the data in Figure 3A. Interestingly, cdk4 was not detectable in the LTNP high abundant individual samples, indicating that this protein was effectively isolated away from the high abundant proteins. Although through a straight western blot, the levels of p16^INK4A ^were undetectable, Figure 3C indicates that p16^INK4A ^can indeed be immunoprecipitated out of the individual low abundant LTNP samples and subsequently detected by western blot. Lanes 1, 2, and 3 represent the pooled "A" sample and patient samples 2 and 3, respectively. The pooled "A" sample was incubated with α-IgG, and patient samples 2 and 3 were incubated with α-p16. These three immunoprecipitations were subjected to very stringent wash conditions of TNE_600 _+ 0.1% NP-40, TNE_300 _+ 0.1% NP-40, and TNE_50 _+ 0.1% NP-40 in order to remove any non-specific proteins. As can be seen in Figure 3C, lanes 1-3 there is some non-specific p16 binding to the α-IgG negative control lane, however, the IPs from the two samples result in much higher percentage of p16^INK4A ^present, especially in lane 3. As compared to lanes 4-10, where the salt washes were of less stringency, there are variable levels of p16^INK4A ^IPed from each patient sample. Based on the background levels of p16^INK4A ^in lane 1, we feel confident in concluding that the patients 2, 3, 4, and 6 have a detectable level of p16^INK4A ^only after immunoprecipitation.

### RT activity of HIV-1 infected cells decreases *in vitro *in the presence of exogenous p16^INK4A^

**Figure 4:**
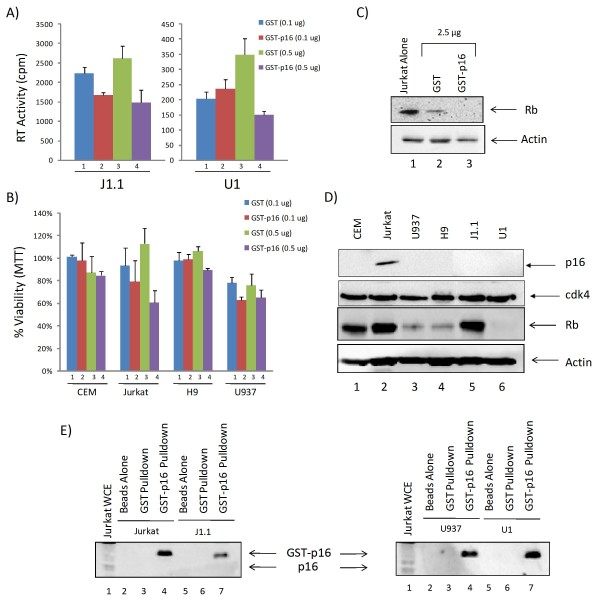
**Viral Replication and Cell Viability assays in the presence of exogenous p16^INK4A ^treatment**. **A) **J1.1 and U1 latently infected HIV-1 cell lines were treated with GST or GST-p16^INK4A ^(0.1 μg or 0.5 μg). Cell culture supernatants were collected at 48 hours post treatment and assayed for reverse transcriptase (RT) activity measured in cpm. **B) **CEM, Jurkat, H9, and U937 uninfected cell lines were treated with GST or GST-p16^INK4A ^(0.1 μg or 0.5 μg). Fourty-eight hours post treatment, the cells were measured for viability with MTT reagent. **C) **Jurkat uninfected T cells were treated with an excess (2.5 μg) of GST or GST-p16^INK4A^. Cells were collected at 48 hours post treatment and western blotted for the presence of Rb and Actin. **D) **CEM, Jurkat, H9, and U937 uninfected cell lines, as well as, J1.1 and U1 latently infected HIV-1 cell lines were assayed for the presence of endogenous levels of p16^INK4A^, cdk4, and Rb. One hundred micrograms of whole cell extract from each cell line was probed with antibodies against p16^INK4A^, cdk4, Rb, and Actin using Western blots. **E) **Uninfected/Infected cell line pairs Jurkat/J1.1 and U937/U1 were treated with GST or GST-p16^INK4A ^(0.5 μg) to test for entry of GST-p16 into the cells. Cells were collected at 48 hours post treatment, washed, lysed, and incubated with Glutathione-Sepharose beads overnight. Beads were washed extensively, and probed for the presence of GST-p16^INK4A ^by western blot against p16^INK4A^. Arrows indicate the endogenously expressed p16^INK4A ^in the control Jurkat lane as well as the larger, GST-p16^INK4A ^band.

Although we have identified p16^INK4A ^as differentially present in the serum of HIV-1 infected LTNPs as compared to HAART treated individuals, this may not directly correlate to viral pathogenesis or functionality of this protein. In order to gain insight into the reason why p16^INK4A ^may be present preferentially in the serum of LTNP patients, we treated latently infected HIV-1 cell lines (J1.1 and U1) with exogenous purified GST-p16^INK4A ^Figure 4A depicts an RT assay which measures the viral reverse transcriptase activity of infected cells and is an indicator of functional particle production. In the presence of both 0.1 and 0.5 ug of GST-p16^INK4A^, J1.1 latently infected T-cells exhibited a decrease in RT activity (cpm) whereas the higher concentration of GST-p16^INK4A ^was able to elicit a decrease in RT activity in the latently infected monocytes, U1, as compared to the GST treatment alone. This data suggests that the presence of p16^INK4A ^in serum may result in a decrease in viral replication, which may help to explain why the presence of p16^INK4A ^in the serum of LTNPs could correlate with an overall lack of viral activity.

### Treatment of uninfected cells with p16^INK4A ^does not affect cellular viability

To detect whether p16^INK4A ^had an effect on normal or uninfected cells, we performed an MTT assay to screen for the percentage of cells viable after p16^INK4A ^treatment. Figure 4B depicts CEM, Jurkat, and H9 uninfected T-cell lines, as well as the uninfected monocytic U937 cell line treated with GST as well as GST-p16^INK4A^. CEM, H9, and U937 control cells showed no appreciable decrease in cellular viability upon 48 hours of treatment with any of the four conditions. Interestingly, in the presence of 0.5 ug of GST-p16^INK4A^, Jurkat cells exhibit an almost 40% decrease in cellular viability. We next performed western blots on whole cell extracts from all four of these uninfected cell lines and observed only Jurkat cells exhibiting an endogenous expression of p16^INK4A ^(Figure 4D, lane 2). This suggests that the decrease in cellular viability seen in Jurkat cells (Figure 4B) treated with p16 ^INK4A ^can be correlated with the expression of exogenous p16^INK4A ^in these cells, resulting in an increase in cdk4,6/Cyclin D inhibition and an increase in apoptosis. Interestingly, no endogenous levels of p16^INK4A ^are detected in the infected J1.1 cells.

### Cellular Rb levels decrease as a result of the exogenous addition of p16^INK4A ^to Jurkat T cells

P16^INK4A ^is a critical member of the Rb tumor-suppressor pathway which acts to arrest the cell-cycle at G1/S by inhibiting the binding of cdk4/6 to Cyclin D1 and subsequently inhibiting the phosphorylation of Rb. In Figure 4C, we investigate the levels of Rb present in Jurkat cells alone (lane 1) compared to Jurkat cells treated with an excess of GST or GST-p16^INK4A ^(2.5 μg, lanes 2 and 3). Interestingly, upon treatment of exogenous GST-p16^INK4A^, we observed a decrease in cellular levels of Rb; indeed there is also a decrease in Rb with GST treatment alone The Rb antibody used detects total Rb levels in the cell, therefore we could not assume a loss of phosphorylation due to the inhibitory effect of p16^INK4A ^on cdk4/6. A recent paper has addressed the literature-wide discrepancies of RB dephosphorylation vs. degradation in response to drug treatment or cell senescence in various cell types [51]. It is possible that the increased amount of p16^INK4A ^present in these cells has induced a proteasomal degradation of Rb that has not otherwise been characterized in T cells. The cell line panel in Figure 4D was also screened for the presence of endogenous levels of Rb in these cell lines, and interestingly there is a high degree of variability. The T cell lines CEM, Jurkat, and the HIV-1 infected J1.1 have the highest endogenous levels of Rb. Interestingly, the monocytic cell lines U937 and the HIV-1 infected U1 have the lowest amount of Rb present, with almost completely undetectable levels in HIV-1 infected U1 cells. The variability supports the discrepancies seen in the literature about hypo-, hyper-phosphorylation of Rb, as well as depletion or degradation of Rb during cell cycle or cellular responses.

### Purified GST-p16 is found intracellularly in Jurkat, J1.1, U937, and U1 after treatment

In order to confirm that the effects seen by GST and GST-p16^INK4A ^treatment in Figure 4A, B, and C, we checked to ensure that the purified proteins are actually entering the cell. Jurkat, J1.1, U937, and U1 cell lines were treated with an excess (2.5 μg) of GST or GST-p16 (Figure 4E). At 48 hours post treatment, the cells were harvested, washed extensively, lysed, and incubated with Glutathione-Sepharose beads overnight. The Glutathione-Sepharose beads were washed extensively to remove any non-specific proteins with buffers containing salts and detergents. The bound proteins were subjected to Western blot for the presence of p16^INK4A ^as shown in Figure 4E. Jurkat whole cell extract served as the positive control (lanes1 in both blots) and a higher molecular weight band corresponding to GST-p16^INK4a ^was observed in the GST-p16^INK4A ^pulldown lanes for each of the cell lines (lanes 4 and 7 in both blots). The lack of detection in the untreated cell lysate incubated with beads alone indicates that the protein detected in lanes 5 and 8 are specifically the GST-bound proteins. These studies confirm that the GST proteins are indeed entering the cells when incubated in the extracellular environment.

### Fascaplysin treatment mimics the exogenous p16^INK4A ^treatment

**Figure 5:**
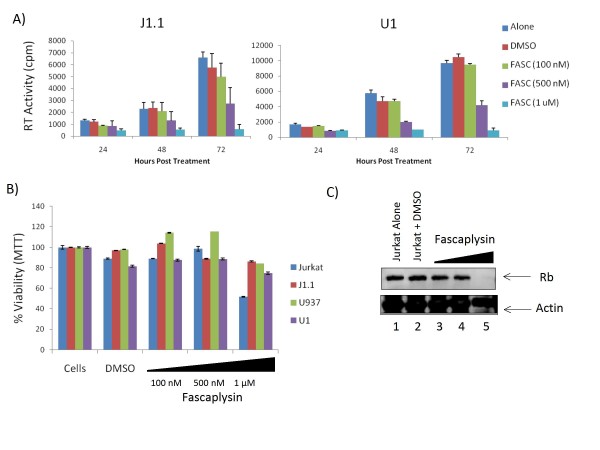
**Viral Replication and Cell Viability assays in the presence of the cdk4/6/Cyclin D inhibitor Fascaplysin**. **A) **J1.1 and U1 latently infected HIV-1 cell lines were treated with Fascaplysin (100 nM, 500 nM, or 1 μM). Cell culture supernatants were collected at 48 hours post treatment and assayed for reverse transcriptase (RT) activity measured in cpm. DMSO treatment served as a negative control. **B) **Jurkat, J1.1, U937, and U1 cell lines were treated with Fascaplysin (100 nM, 500 nM, or 1 μM). Fourty-eight hours post treatment, the cells were measured for viability with MTT reagent. DMSO treatment served as a negative control. **C) **Jurkat uninfected T cells were treated with Fascaplysin (100 nM, 500 nM, or 1 μM). Cells were collected at 48 hours post treatment and western blotted for the presence of Rb and Actin. DMSO treatment served as a negative control.

In order to confirm that the cellular effects shown in Figure 4 are specific to the natural biological activity of p16^INK4A ^as a cdk4/6/Cyclin D inhibitor, we attempted to mimic these studies with the small molecule compound inhibitor Fascaplysin. Fascaplysin (FASC) is a naturally derived molecule isolated from a marine sponge which specifically inhibits the interaction between cdk4/Cyclin D at an IC_50 _of approximately 0.35 μM, and to a lesser extent cdk6/Cyclin D by binding the ATP pocket of cdk4, resulting in cell cycle arrest at G1/S [52,53]. Again, we treated latently infected HIV-1 cell lines (J1.1 and U1) with three concentrations of FASC (100 nM, 500 nM, and 1 μM) and collected supernatants at 24, 48, and 72 hours post treatment. The RT activity of both J1.1 and U1 cells in the presence of FASC decreased over time with increasing concentration of the drug. This indicates that the presence of a general cdk4/Cyclin D inhibitor is able to decrease viral production in the same manner as exogenous p16^INK4A^. Additionally, we performed an MTT assay to screen for the percentage of cells viable after Fascaplysin treatment. Figure 5B depicts % viability of Jurkat, J1.1, U937, and U1cells treated with three concentrations of FASC (100 nM, 500 nM, and 1 μM) after 48 hours. Correlating with the viability assay presented in Figure 4B, approximately 50% of Jurkat cells were killed due to additional cdk4/Cyclin D inhibition by 1 μM of FASC treatment. None of the other cell lines exhibit appreciable cell death which indicates that the drug treatment itself is not toxic to the cells. In Figure 5C, we investigate the levels of Rb present in Jurkat cells alone (lane 1) compared to Jurkat cells that have been treated with three concentrations of FASC (lanes 3, 4, and 5). Again, correlating with our exogenous p16^INK4A ^treatment data in Figure 4, we observed a decrease in total cellular Rb levels at the highest concentration of FASC. This set of data confirms that the cellular effects we observed with exogenous p16^INK4A ^may be due to the specific cdk inhibitory activity of this molecule. It is interesting to note that these effects are seen with simple protein treatment of the cells with a purified molecule which may not have efficient entry as compared to transfection or drug treatment. This suggests that p16^INK4A ^in the serum may be able to enter and exit lymphocytes and exhibit its inhibitory effects during an HIV-1 infection.

### Fascaplysin treatment increases apoptosis in Jurkat cells and arrests latently infected J1.1 cells at G1/S *in vitro*

**Figure 6:**
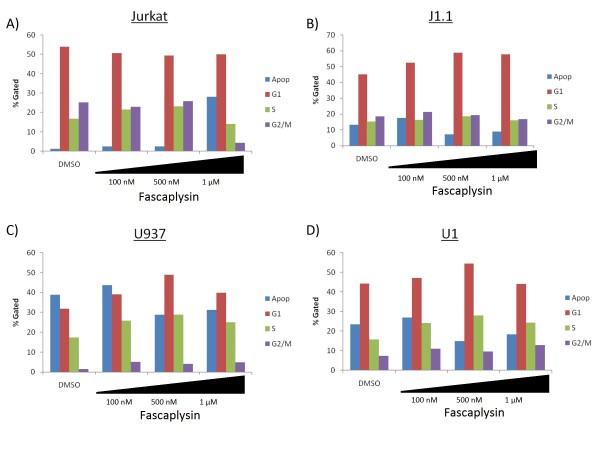
**Fascaplysin induces apoptosis in Jurkat cells and G1/S arrest in latently infected J1.1 cells**. Cell cycle analysis was performed on **A) **Jurkat, **B) **J1.1, **C) **U937, **D) **U1 that were treated with DMSO, 100 nM, 500 nM, or 1 μM Fascaplysin for 48 hours. Bars represent the percentage of gated cells present in each of the cell cycle stages: G1, S, G2/M or those that had apoptosed.

We previously showed that both p16^INK4A ^and Fascaplysin treatment results in a loss of cellular viability in Jurkat cells as well as a decrease in viral production in infected J1.1 and U1 cells. We were interested to detect the cell cycle pattern of Jurkat, J1.1, U937, and U1 in response to Fascaplysin treatment. Cells were treated with three concentrations of FASC (100 nM, 500 nM, 1 μM) and were collected after 48 hours. Cells were fixed and stained with Propidium Iodide and cell cycle analyzed using a FacsCalibur Flow Cytometer. In Figure 6A-D, we compare the population of cells in each stage of the cell cycle at the three concentrations of FASC in Jurkat, J1.1, U937, and U1 cells, as compared to the DMSO control. At the highest concentration of FASC, we observe an increase in the apoptotic peak in Jurkat cells alone. This correlates with the cellular viability data in p16^INK4A ^and FASC treated cells. Interestingly, we observed an arrest of cells at G1 in the all of the other treated cell lines. These cell lines do not contain endogenous levels of p16^INK4A^, therefore in the presence of an additional cdk4/6/Cyclin D inhibitor, we observed the normal G1/S arrest effect. There were no major appreciable differences in cell cycle in the FASC treated U937 and U1, however, looking at the protein profile of endogenously expressed p16^INK4A ^and Rb, it is not surprising that these monocytic cell lines would exhibit a different inhibitory pathway. Taken together, the cell cycle data shown in Figure 6, supports the overall notion of cdk4/6/Cyclin D inhibitory effect by p16^INK4A ^and Fascaplysin.

## Discussion

The global proteomic analysis of serum proteins is not without its challenges; however the presence or absence of proteins in such bodily fluids of patients is often the most accurate reflection of cellular leakage or secretion of proteins in response to a disease state. Unfortunately, the classical serum proteome contains a large concentration of high abundance proteins that mask the individual proteins that are unique to a particular phenotype. The identification of such low abundance serum proteins is attractive as a method of early detection of a disease state or a response to infection. Here, we demonstrate that the detection of unique proteins in the serum of HIV-1 infected long-term non-progressors may be indicative of a natural "immunity" to the progression of HIV-1 infection. Specifically, we have identified p16^INK4A^, a cdk4/6 inhibitor, as preferentially present in pooled serum of HIV-1 LTNP patients, as opposed to HIV-1 infected individuals responding to HAART treatment.

P16^INK4A ^is a member of the INK4/ARF family of endogenous cdki's that serve to regulate cell cycle progression through the inhibition of specific cdk/Cyclin interactions. P16^INK4A ^is a critical member of the Rb/p16 tumor-suppressor pathway which inhibits the activation of cdk4/6, preventing the progression through the cell cycle. This tumor-suppressive pathway is mutated in close to 100% of human cancers and specific loss-of-function mutations are found within the *Rb *gene or the *CDKN2A *gene encoding p16^INK4A ^[54]. In addition to the direct inactivation of these two proteins, cancer cells also override this regulatory pathway by overexpressing cdk/Cyclins as well as inducing the endogenous loss of expression of other cdk inhibitors. The loss of p16^INK4A ^results in the constitutive activation of cdk4/6 as well as pRb hyperphosphorylation, therefore bypassing the anti-oncogenic senescence induced by this cdk inhibitor. In addition to necessitating an oncogenic state, the regulation and manipulation of cellular cdks and Cyclins is also critical for an active HIV-1 viral infection and occurs mainly through the HIV-1 transactivator Tat. Tat interacts with the cdk2/Cyclin E complex to phosphorylate cdk7 and assist in the phosphorylation of the C-terminal Domain (CTD) of RNA Pol II at the viral LTR (long-terminal repeat) to promote viral transcription [55-57]. Cdk2 is also involved in the direct phosphorylation of Tat at both serine 16 and 46, which are critical for efficient Tat activity [56,57]. HIV-1 transactivation is also heavily dependent on the recruitment of the cdk9/Cyclin T1 complex to the viral TAR element where it also assists in the phosphorylation of the CTD of RNA Pol II as well as an autophosphorylation event which is important for the localization to the nucleus [58]. Taking into consideration the recruitment of cellular kinases for HIV-1 transcription, it is not surprising that pharmacological cdk inhibitors have been developed (analogous to endogenous cdki's) as a template to specifically inhibit these kinases. Therefore, the presence of the cdk inhibitor p16^INK4A ^in LTNP serum is suggestive of an existing defense mechanism in these patients that imparts a predisposition to a lack of HIV-1 disease progression.

The development of pharmacological cdki's and peptide mimetics is a commonly used approach for both cancer and viral therapeutics. For instance, p16^INK4A ^peptides have previously been developed, which, when conjugated to localization proteins/peptides, were able to block cell cycle progression in breast cancer and colon cancer cell lines *in vitro *as well as reduce tumor size in an *in vivo *mouse model of pancreatic cancer [59-61]. These peptides have also been used for intracellular delivery to leukemia and lymphoma derived cells [59,62]. These studies have demonstrated the ability of cdki-derived peptides to be used for both direct cell cycle arrest and the treatment of metastatic cancers. This type of therapy is an appropriate approach to cancerous states where the oncogenic mutation results in a loss of function or expression of the p16^INK4A ^protein. Here, we show that p16^INK4A ^is only endogenously expressed in the Jurkat T-cell line and additional treatment of Jurkat cells with exogenous p16^INK4A ^results in a decrease in cellular viability. All additional uninfected and infected cell lines used in this study did not express endogenous levels of p16^INK4A^, suggesting that a loss of function or expression may be present in these particular cell types. More importantly, the treatment of stably infected HIV-1 cell lines, J1.1 and U1, with exogenous p16^INK4A ^resulted in a decrease in viral replication with a minimal decrease in cellular viability, suggesting that the presence of p16^INK4A ^in the context of an *in vitro *HIV-1 infection may mimic an *in vivo *infection in a LTNP patient. The same dosage of p16 ^INK4A ^needed for a decrease in viral replication in infected cells is not toxic to uninfected cells, with the exception of Jurkat cells, indicating that there may be a threshold for which the effect of this cdk inhibitor begins to harm the cell and potentially induce cell cycle arrest and apoptosis.

In this study we also chose to reinforce the p16^INK4A^-induced cdki effects seen on cellular viability and viral replication with a known cdk4/6/Cyclin D inhibitor, Fascaplysin. We show that treatment of Jurkat cells with this inhibitor also results in a decrease in cellular viability, consistent with the effect seen by treatment with endogenous p16^INK4A^. Additionally, the treatment of both stably infected HIV-1 cell lines, J1.1 and U1, with Fascaplysin resulted in a decrease in viral replication with a minimal decrease in cellular viability. The correlation of this pharmacological cdki inhibitor data with the functional-cdki p16^INK4A ^data suggests that the preferential inhibition of the cdk4/6/Cyclin D interaction by LTNPs may contribute to this phenotype.

The high and low abundance fractions analyzed for each of the three patient types were a pooled representation of six individual patient samples that almost certainly have inherent variability. Indeed, when assaying for p16^INK4A ^in high and low abundance serum fractions for each individual LTNP patient, p16^INK4A ^was present in only a subset of the patients analyzed. This indicates that although p16^INK4A ^may serve as a unique serum protein indicating a LTNP HIV-1 infected status, individual person-to-person changes will alter the serum proteome profile and needs to be taken into consideration. Differences in expression of p16^INK4A ^can be due to either a host cellular response to the infection or from an influence of the infection itself. Two different scenarios need to be considered when examining the potential importance of this protein in defining a viral disease state such as a LTNP patient; the effect on the host and the effect on the virus.

Finally, many complicating factors can contribute to the altered state of proteins present in the serum, not the least of which is the length of time in which the patient has been infected, age, gender, coinfections, and other preexisting conditions such as cancer or metabolic diseases. Therefore, it is important to take into consideration some of these factors when applying global proteomic analyses to HIV-1 patient derived samples.

## Materials and methods

### Cell Culture and Protein Reagents

293T endothelial kidney cell line was harvested for whole cell extract and used as a positive control. 293Ts were grown in Dulbecco's modified Eagle's medium (DMEM) containing 10% FBS, 1% L-glutamine, and 1% streptomycin/penicillin (Quality Biological). Latently infected HIV-1 cell lines J1.1 (T-cells) and U1 (monocytes) were used for RT assays and whole cell extracts were obtained for Western blots. Uninfected CEM, Jurkat, and H9 cell lines (T-cells) and uninfected U937 cell line (monocytes) were used for viability assays and whole cell extracts were obtained for Western blots. Uninfected cells were grown in RPMI-1640 media containing 10% FBS, 1% L-glutamine, and 1% streptomycin/penicillin (Quality Biological). All cells were incubated at 37°C and 5% CO2. GST-p16^INK4A ^was a generous gift from Dr. Ming-Daw Tsai, Institute of Biological Chemistry, Academia Sinica, Nankang, Taipei, Taiwan

### Serum Samples and Serum Depletion

Eighteen subject serum samples (6 LTNP, 6 HIV infected subjects receiving HAART therapy, and 6 uninfected individuals) were obtained through Washington DC site of the Women's Interagency HIV Study (WIHS) (Table 1). WIHS is an NIH multicenter study of the natural history of HIV-1 infection in women [29]. LTNPs were defined by WIHS as being HIV Infected, but disease free for at least five years, a CD4 count of greater than 500 at all visits, and no history of anti-retroviral therapy. Serum samples were subjected to depletion of the 12 most abundant serum proteins using the ProteomeLab IgY-12 High Capacity Spin Column Proteome Partitioning kit from Phenomenex (Torrance, CA). This spin column consists of anti-human serum albumin, anti-IgG, anti-fibrinogen, anti-transferrin, anti-IgA, anti-IgM, anti-HDL (anti-apo A-I and anti-apo A-II), anti-haptoglobin, anti-α1-antitrypsin, anti-α1-acid glycoprotein and anti-α2-macroglobulin conjugated to polymeric microbeads. Twenty microliters of each serum sample was diluted 1:25 in dilution buffer and ran over the spin column. Low abundant proteins were collected in the flowthrough and subsequent high abundant, bound proteins were retained and eluted with a low pH stripping buffer. Protein concentrations of both low and high abundant fractions were calculated and pooled as indicated.

### 2D-Gel Electrophoresis (2DGE) and MALDI-TOF MS

Five hundred micrograms of pooled LTNP, HAART, and Negative patient samples for both Low and High abundance fractions were subjected to isoelectric focusing on an IPG strip, pH 3.0-10.0 and further subjected to SDS-PAGE on a 4-20% Criterion Tris-Glycine gel. The gels were stained with Coomassie Blue and protein spots of interest were excised. Spots were vortexed, washed, and equilibrated in 25 mM NH_4_HCO_3_, broken into smaller pieces, and vortexed and washed 3X with 50% ACN/25 mM NH_4_HCO_3 _to remove the Coomassie from the gel. Gel pieces were vortexed and washed with 100% ACN to dehydrate the gel pieces. Gel pieces were re-swelled with up to 200 ng of trypsin (in enough volume to cover the gel) and incubated on ice for 30 min. Residual trypsin was removed, 20 μl of 25 mM NH_4_HCO_3 _or enough to cover the gel was added to the gel pieces and the reactions were incubated overnight at 37°C. Peptides were extracted with 1X dH_2_O wash with brief vortexing and sonication, followed by 3X washes with 60% ACN/5% TFA. Extracted peptides were pooled together and a SpeedVac was utilized to reduce the volume to approximately 10 μl. Twenty microliters of 0.1% TFA was added to each tube and peptides were desalted using C_18 _ZipTips (Millipore) according to manufacturer's instructions. Peptides were spotted on MALDI sample plate 1:1 with α-cyano-4-hydroxy cinnamic acid (CHCA) matrix solution: 10 mg CHCA, 500 μl 100% ACN, 500 μl 0.1% TFA. Positive control calibration peptide solution of Bradykinin, Angiotensin II, P_14_R, and ACTH was spotted along with negative control empty gel slice. Mass peaks obtained were entered into Mascot http://www.matrixscience.com/ and ProFound http://prowl.rockefeller.edu/prowl-cgi/profound.exe databases for peptide mass fingerprinting analysis.

### Immunoprecipitations

Fifty microliters of pooled low abundance LTNP serum sample "A" and individual low abundance LTNP serum samples (1-6) were incubated with either α-IgG or α-p16^INK4A ^as indicated, volume was brought up to 500 μl with TNE_50 _+ 0.1% NP-40, rotating overnight at 4°C. Extracts were incubated with 50 μl of a 30% slurry of Protein A + G Agarose beads (Calbiochem #IP05) for 2 hrs, rotating, at 4°C. Beads were washed with indicated salt washes (TNE_600 _+ 0.1% NP-40, TNE_300 _+ 0.1% NP-40, TNE_150 _+ 0.1% NP-40, or TNE_50 _+ 0.1% NP-40), bound proteins were removed from the beads with Laemmli buffer and subjected to Western blots against p16^INK4A^.

### Western Blots

Western blots were performed to validate proteins identified from depleted serum samples. Whole cell extracts were obtained from cell culture pellets washed twice with 25 mL of phosphate buffered saline (PBS) with Ca2+ and Mg2+ (Quality Biological) and centrifuged once more. Cell pellets were resuspended in lysis buffer (50 mM Tris-HCl, pH 7.5, 120 mM NaCl, 5 mM EDTA, 0.5% NP-40, 50 mM NaF, 0.2 mM Na3VO4, 1 mM DTT, one complete protease cocktail tablet/50 mL) and incubated on ice for 20 min, with a gently vortexing every 5 min. Cell lysates were transferred to eppendorf tubes and were centrifuged at 10,000 rpm for 10 min. Supernatants were transferred to a fresh tube where protein concentrations were determined using Bio-Rad protein assay (Bio-Rad, Hercules, CA). Antibodies against cdk4 (sc-749), p16 (sc-467), PCTAIRE (sc-174), Rb (sc-50), and Actin (sc-1615) were purchased from Santa Cruz Biotechnology (Santa Cruz, CA). Antibodies against HP1α and HP1γ were purchased from Cell Signaling (Danvers, MA).

### RT Assays

J1.1 and U1 cells (2 × 10^6^) were treated with GST or GST-p16 (0.1 or 0.5 μg) or Fascaplysin (100 nM, 500 nM, or 1 μM) and supernatants collected to test for the presence of virus 48 hours post treatment. Jurkats were treated with GST or GST-p16 (2.5 μg) for 48 hours prior to HIV-1 infection with the dual tropic strain 89.6. Supernatants were collected at various time points post infection to test for the presence of virus post treatment. Viral supernatants (10 μl) were incubated in a 96-well plate with reverse transcriptase (RT) reaction mixture containing 1X RT buffer (50 mM Tris-HCl, 1 mM DTT, 5 mM MgCl_2_, 20 mM KCl), 0.1% Triton, poly(A) (1U/ml), pd(T) (1U/ml), and [3H]TTP. The mixture was incubated overnight at 37°C, and 5 μl of the reaction mix was spotted on a DEAE Filtermat paper, washed four times with 5% Na_2_HPO_4_, three times with water, and then dried completely. RT activity was measured in a Betaplate counter (Wallac, Gaithersburg, MD).

### MTT Assays

Five thousand cells were plated per well in a 96-well plate and the next day cells were treated with GST or GST-p16 (0.1 or 0.5 μg) or Fascaplysin (100 nM, 500 nM, or 1 μM). Forty-eight hours later, 10 μl MTT reagent (5 mg/ml) was added to each well and plates incubated at 37°C for 3 hours. Next, 100 μl of DMSO was added to each well to solubilize the violet crystals. The assay was read at 570 nM.

### Cell cycle analysis

Cells were washed with PBS and fixed with 70% ethanol. Following rehydration in PBS, cells were stained in PBS containing 25 ug/ml propidium iodide (Sigma), 10 ug/ml RNase A (Sigma) and 0.1% NP-40. Cells were analyzed on a BD FacsCalibur flow cytometer. Cell cycle analysis and measurement of apoptosis was performed using CellQuest software. Aggregates and debris were excluded by gating on the FL2W and FL2A parameters. Apoptosis was considered to be the population of cells that were sub-G1.

## Competing interests

The authors declare that they have no competing interests.

## Authors' contributions

RVD performed the serum depletion, 2D gel analysis, MALDI preparation, and mass spectrometry analysis as well as the drafting of the manuscript. IG performed serum depletion, MALDI preparation, and confirmatory western blots. KKH performed the RT and MTT assays. RE performed the MALDI preparation and analysis. ZK provided support in drafting the manuscript and MALDI analysis. CL established the patient definitions (LTNP, HAART responders, etc.) and patient sample identification. MY is the WIHS contact and provided support and information on patient samples. FK provided overall direction and funding for the project. All authors have read and approved the final manuscript.
